# Design of an Optical Transparent Absorber and Defect Diagnostics Analysis Based on Near-Field Measurement

**DOI:** 10.3390/s21093076

**Published:** 2021-04-28

**Authors:** In-Gon Lee, Young-Joon Yoon, Kwang-Sik Choi, Ic-Pyo Hong

**Affiliations:** 1 Information and Communication Engineering Department, Kongju National University, Cheonan 31080, Korea; igl38@kongju.ac.kr; 2 Korea Institute of Ceramic Engineering & Technology, Jinju 52851, Korea; yjyoon@kicet.re.kr; 3 Korea Aerospace Industries, Sacheon 52529, Korea; kwangsik.choi@koreaaero.com

**Keywords:** radar absorbing structure, transparent conductive oxide, defect diagnostics, near-field measurement

## Abstract

To reduce the electromagnetic wave interference caused by cavity resonance or electromagnetic wave leakage, herein, an optical transparent radar absorbing structure (RAS) was designed using transparent conductive oxides (TCOs) with a high optical transmittance and electrical conductivity, and a procedure was proposed for detecting possible defects in the fabrication and operation and for assessing the influence of the defects on the electromagnetic performance. To detect locally occurring defects in planar and three-dimensional absorbing structures, a measurement system based on an open-ended near-field antenna capable of producing small beam spots at a close distance was constructed. Moreover, the reflection characteristics of the transparent RAS were derived from a simplified multiple reflection equation, and the derived results were compared with the analysis results of an equivalent circuit model to predict the surface resistance of the TCO coating layer, based on which the presence of defects could be confirmed. By using the experimental results, the predicted surface resistance results of the coating layer and the results measured using a non-contact sheet resistance meter were compared and were found to correspond, thereby confirming the effectiveness of the proposed defect detection method.

## 1. Introduction

Radar absorbing materials that convert electromagnetic wave incidents from any direction to thermal energy and then absorb them, and radar absorbing structures (RAS) that cancel waves through interference are used in various fields such as radar cross-section (RCS) reduction technology [1,2,3,4]. The RCS reduction technology is one of the most important stealth technologies in defense, and is applied as interference reduction technology for improving communication performance in complex wireless communication environments in the private sector [5,6,7,8,9,10,11]. Researchers are continuously investigating techniques such as those using Dallenbach layers, Salisbury screens [5,6], and circuit analog absorbers [8,9,10,11]. These are resonant electromagnetic structures that maximize return loss by inducing destructive interference in a specific frequency band on electromagnetic wave incidents through a dielectric and resistive surface. Most research on RASs focuses on materials based on magnetic substances such as polymer ferrite or conductive materials such as carbon nanotubes and metal fibers in the form of paints, coatings, and lossy dielectrics (conductive/magnetic, ceramic polymer composites) [1,12,13]. There has been limited research on designing RASs based on transparent materials. Absorbing structures based on transparent conductors and materials have been developed recently. Typically, for the design and fabrication, a transparent conductor, such as indium-oxide-based transparent conductive oxide (TCO), aluminum-doped zinc oxide, or fluorine-doped tin oxide, is coated on a transparent material [7,8,9,10,14], or a gold or silver mesh with a line width of 15 nm or less is printed [11,15,16]. However, these studies focused on obtaining small-size transparent RASs and achieving an excellent electromagnetic and optical performance [7,8,11]. Although they facilitate ease-of-design via the use of a TCO-coated film through laser lithography, it is difficult for them to realize a large area. Hence, their practical applications are limited [9,10]. There is insufficient research on detecting the defects (microcracks, vacancy defects, and non-uniform surface resistance) that can occur during the fabrication and operation of large areas or three-dimensional structures, as well as investigating the influence of the defects on the electromagnetic performance. In this study, Dallenbach and Salisbury structures were designed and fabricated using a low-temperature magnetron sputter-deposition process, which can fabricate large-area three-dimensional structures and easily realize various surface resistance characteristics. These structures block interference through the cavity effect caused by structural resonance and selectively absorb specific frequency bands. The reflection/transmission and absorption characteristics based on free-space measurement (FSM) were then evaluated to verify the effectiveness of the design.

**Figure 1:**
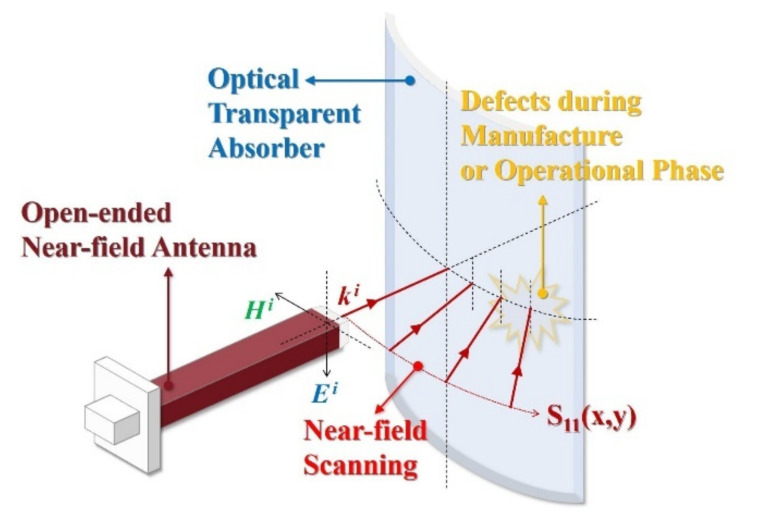
Illustration of the evaluation scenario for an optical transparent absorber with a three-dimensional structure.

Furthermore, to detect the defects that can occur during fabrication and operation, and to determine their influence (as shown in Figure 1), a measurement system was constructed using an open-ended near-field antenna capable of measuring the electromagnetic performance for a local area at a close distance. Moreover, the results were verified by a defect detection algorithm based on an equivalent circuit-analysis model, which demonstrated that the proposed defect analysis scenario and procedure could be practically applied as a type of nondestructive testing method.

This paper consists of four sections. Section 2 describes the design and fabrication of the transparent absorber, and the evaluation of the absorber’s reflection/transmission and absorption characteristics using FSM. Section 3 describes the defect detection procedure based on the equivalent circuit model, the algorithm design, and the feasibility of the study results for the defect detection algorithm. Finally, Section 4 presents the conclusions and contributions of this study.

## 2. Design, Fabrication, and Measurement of an Optical Transparent Absorber

### 2.1. Design of an Optical Transparent Absorber

A Dallenbach structure coated with TCO possessing a low surface resistance, or a Salisbury structure with frequency-selective absorption characteristics for hot spot areas, where many electromagnetic waves are reflected in a specific direction, can be effective at blocking interference because of the electromagnetic wave leakage from the inside of the structure and via the cavity effect caused by a structure composed of materials with high radio wave reflection characteristics, as well as from the electromagnetic wave incidents from the outside.

**Figure 2:**
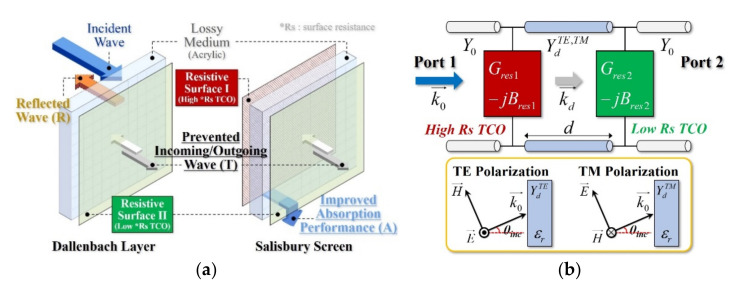
The configuration of the optical transparent absorber and equivalent circuit model: (**a**) structure of a transparent conductive oxide (TCO) coated acrylic absorber and an (**b**) equivalent circuit model.

A film of indium-oxide-based TCO, which can easily obtain a low sheet resistance and secure high visible light transmittance characteristics, was applied on an acrylic substrate. Figure 2a shows the transparent RAS in which TCO is coated on one or both sides; Figure 2b shows the equivalent circuit model. An acrylic substrate was used for the transparent dielectric; for the dielectric constant, the values of ε_r_ = 2.55 and tanδ = 0.0052 measured with FSM for the X-band were used.

The radio wave transmission characteristics of the transparent RAS were proportional to the surface resistance of the coated conductive film. By configuring a spacer with a thickness that is one-fourth the wavelength of the center frequency of the absorption band, as well as with high- and low-resistance surfaces, the radio wave absorption characteristics could be realized. This could be expressed as an ABCD matrix, as in Equation (1), from the equivalent circuit shown in Figure 2b, and the reflection and transmission characteristics could be calculated using Equations (2) and (3) [17]. In Equation (1), $Y_{d}^{TE}=\left(\omega \mu_{r}\mu_{0}\right)/k_{d}$, $Y_{d}^{TM}=k_{d}/\left(\omega \varepsilon_{r}\varepsilon_{0}\right)$ are characteristic admittance of the dielectric substrate for TE and TM polarization under the oblique incidence, where $k_{d}=\sqrt{\varepsilon_{r}k_{0}^{2}-k_{0}^{2}\sin^{2}\theta_{inc}}$ is the normal component of the wavenumber. $\varepsilon_{0}$ and $\mu_{0}$ are the permittivity and permeability of free space, respectively, and $\varepsilon_{r}$ and $\mu_{r}$ are the relative permittivity and permeability of the medium. The propagation constant of the free space is $k_{0}$ and the angle of incident wave is denoted as $\theta_{inc}$. The ideal Dallenbach layer and Salisbury screen can be modeled by $Y_{res1}\approx 0$, $Y_{res2}\approx \infty$ and $Y_{res1}\approx Y_{0}$, $Y_{res2}\approx \infty$, respectively.

For the transmission characteristics, the admittance of the low-resistance surface ($Y_{res2}$) acts as a design variable with considerable influence. Moreover, for the radio wave absorption characteristics, the same is true for the admittance of the high-resistance surface ($Y_{res1}$) and the spacer thickness d.

(1) $$\left[\begin{array}{cc} A & B\\ C & D \end{array}\right]=\left[\begin{array}{cc} 1 & 0\\ Y_{res1} & 1 \end{array}\right]\left[\begin{array}{cc} \cos k_{d}d & j\sin k_{d}d/Y_{d}^{TE,TM}\\ jY_{d}^{TE,TM}\sin k_{d}d & \cos k_{d}d \end{array}\right]\left[\begin{array}{cc} 1 & 0\\ Y_{res2} & 1 \end{array}\right]$$

(2) $$\left|S_{11}\right|=\left|\frac{A+BY_{0}-C/Y_{0}-D}{A+BY_{0}+C/Y_{0}+D}\right|$$

(3) $$\left|S_{21}\right|=\left|\frac{2}{A+BY_{0}+C/Y_{0}+D}\right|$$

In order to obtain the admittance characteristics of the high-resistance surface so as to realize the optimal absorption condition ($\left|S_{11}\right|=0$) in a specific frequency band, assuming that the high-resistance surface admittance is $Y_{res1}=G_{res1}$ (pure conductance), the low-resistance surface has total reflection ($Y_{res2}\to \infty$) characteristics, and the characteristic impedance of the spacer is equal to that of free space ($Y_{d}=Y_{0}=1/120\pi$), then the admittance of the high-resistance surface to realize the optimal absorption condition has the relationship shown in Equation (4).

(4) $$G_{res1}=Y_{0},B_{res1}=-Y_{0}\cos k_{d}d/\sin k_{d}d$$

From Equation (4), the real part of the high-resistance surface that determines the absorption performance as a parameter for realizing the optimal absorption condition is the characteristic admittance of free space. Moreover, for the imaginary part, when the spacer thickness is an odd multiple (n) of one-fourth the wavelength of the operating frequency, the phase delay of the resistive surface becomes zero and the waves perfectly cancel out because of the reflected phase difference.

### 2.2. Fabrication and Measurement

**Figure 3:**
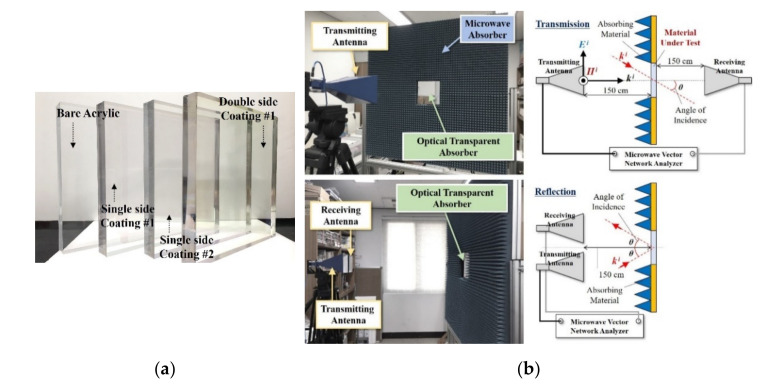
The fabricated optical transparent absorber and measurement environment for evaluating the electromagnetic wave transmission/reflection characteristics: (**a**) the optical transparent absorber sample and the (**b**) measurement setup.

Considering the application environment based on the analysis results using the equivalent circuit model, transparent absorbing structures were implemented, in which TCO was coated on one or both sides of acrylic dielectrics with a size of 250 mm × 250 mm and thickness (d) of 10 mm and 22.5 mm. With the basic goal of blocking 90% (10% transmittance in terms of power) of the incident electromagnetic waves on the large-area transparent RAS, to realize at least 90% absorption performance in the center frequency band when the surface resistance of the low-resistance structure was 30 Ω/sq or less, and when both sides were coated, the high-resistance surface was designed to have a surface resistance of 360 Ω/sq. The structure was fabricated via DC magnetron sputtering deposition and is shown in Figure 3a.

As shown in Figure 3b, the electromagnetic performance of the fabricated transparent RAS was measured using FSM, and a measurement system was constructed using a jig attached with a pyramidal wave absorber so as to reduce the edge effects in the sample, such as diffraction, as well as two independent horn antennas and a vector network analyzer (VNA). The reflection/transmission characteristics could be measured by the following procedure: (a) the VNA was calibrated with the standards Short, Open, Load, and Thru (SOLT) using a Calibration Kit, and (b) the free-space calibration was performed, where the reference measurements of the reflection could be obtained by measuring the data with a metal plate as a MUT (material under test). In the case of transmission characteristics, the reference measurement could be carried out by measuring the path loss for free space without any MUT.

After the calibration process, the reflection/transmission characteristics were measured using the difference between the input power ($P_{inc}$) applied from the transmission antenna and the power received ($P_{trans}$) through the sample, and the absorption characteristics ($P_{abs}$) were evaluated using Equation (5), based on the measured results.

(5) $$P_{abs}=P_{inc}-P_{reflec}-P_{trans}$$

**Figure 4:**
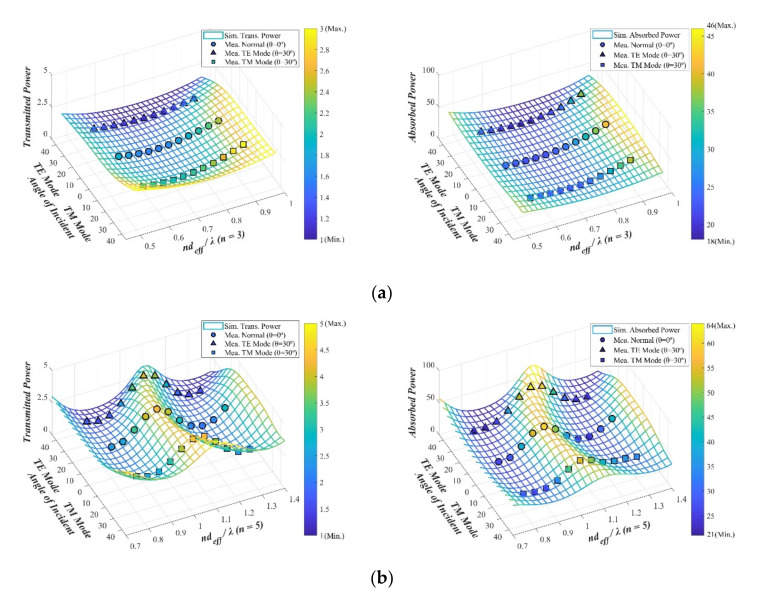

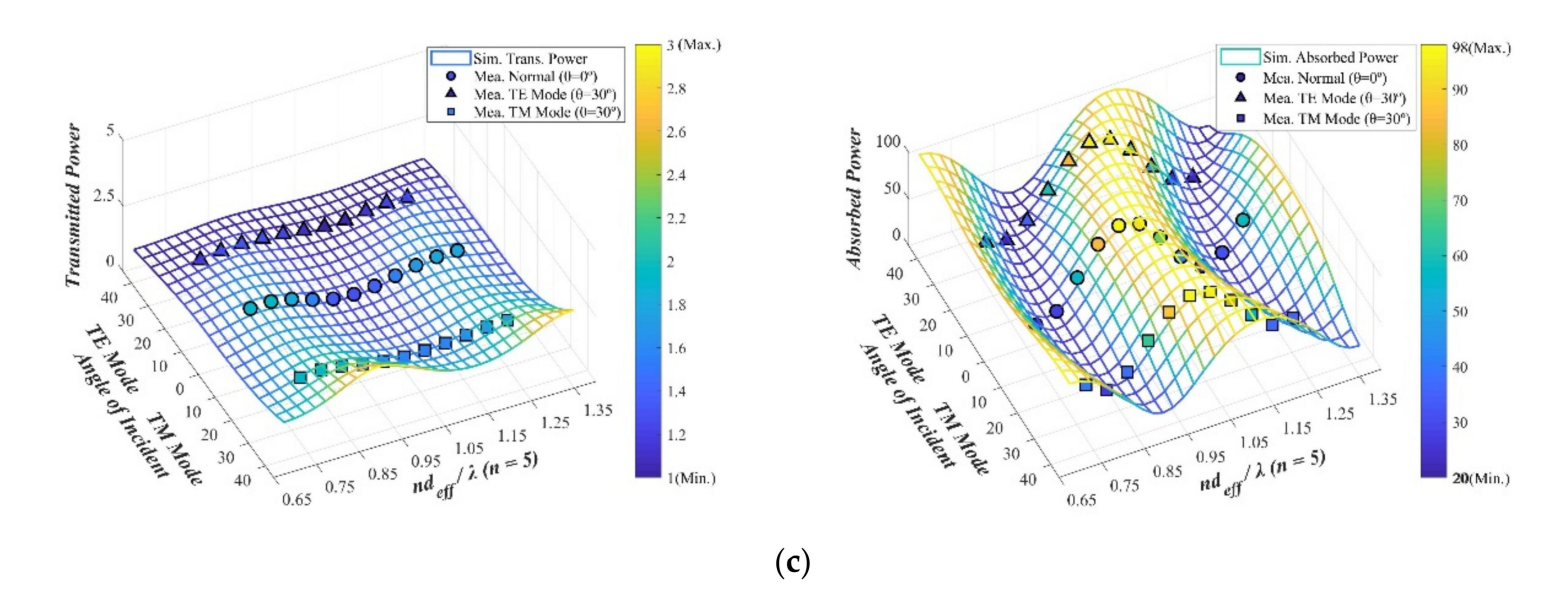
The performance of the fabricated optical transparent absorber for oblique incidence (simulated (Sim.) and measured (Mea.) results for the transmission and absorption characteristic in %): (**a**) sample 1, (**b**) sample 2, and (**c**) sample 3.

Radio wave transmission/absorption characteristics were measured for the 8–12 GHz band with oblique incidence conditions, and the results are shown in Figure 4a–c, where $d_{\mathrm{eff}}=4d\sqrt{\varepsilon_{r}}$ is the effective thickness and λ is the wavelength in the interested frequency band. The measurement results confirmed that the maximum radio wave transmittance was 2.01% under normal incidence conditions for the single-sided (Figure 4a,b) and double-sided (Figure 4c) coating structures. The radio wave absorption was a maximum of 32.3% ($nd_{eff}/\lambda =0.89,\;n=3$) for the single-sided coating structure and a maximum of 98.1% ($nd_{eff}/\lambda =0.96,\;n=5$) for the double-sided coating structure.

## 3. Defect Diagnostics Analysis

### 3.1. Measurement of Reflection Characteristics Based on Single Near-Field Antenna

The physical defects that occur during fabrication and operation must be considered in order to facilitate the practical application of the transparent RAS design. However, because of the nature of the material that exhibits the optical transparency, it is difficult to detect defects with the naked eye and to evaluate how actual defects influence electromagnetic performance. Consequently, it is difficult to determine if repairs or replacements are necessary. To solve these issues, research on nondestructive testing methods suitable for transparent absorbers is necessary. As explained above, the performance of the RAS using TCO is determined by the surface resistance of the resistive surface coated with TCO. The surface resistance value of the coating layer can be obtained, with a high accuracy, using a measuring device such as a non-contact sheet resistance meter.

Moreover, the degree of change in the measured surface resistance can be used to detect defects and to identify the defect area. However, because of the characteristic of the probe, whcih requires perfect contact to measure the surface resistance based on an eddy current measuring method, the applicability of the probe is limited to planar RASs. It is also difficult to accurately measure the surface resistance if the coating layer is non-uniform and rough (e.g., z metal–mesh–grid-based transparent absorber) [8,16].

**Figure 5:**
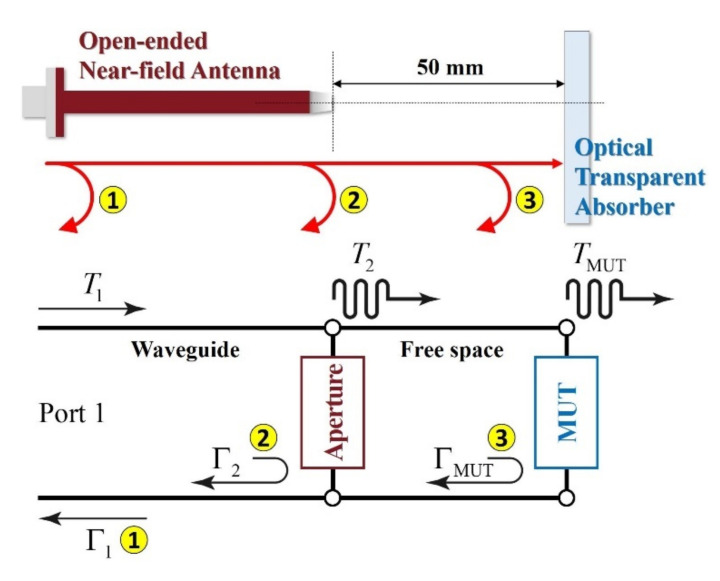
The schematic of measurement setup for reflectivity based on a single near-field antenna.

To solve these problems, in this study, a measurement system was constructed using an open-ended near-field antenna capable of evaluating the electromagnetic performance for a local area at a close distance. To obtain the reflection characteristics of the transparent RAS using only a single antenna, the closed-circuit condition considering the effects of the antenna, free space, and sample needed to be satisfied (Figure 5) [18] based on the definition of the reflection coefficient. From the simplified quarter-wave transformer of Equation (6), for $\Gamma_{3}$, assuming no reflection $\Gamma_{\mathrm{Air}}$(=0), total reflection from the conductive medium $\Gamma_{\mathrm{PEC}}$(=−1), and reflection from the absorber $\Gamma_{\mathrm{MUT}}$, Equation (7) can be expressed as follows. By obtaining $\Gamma_{\mathrm{Air}}$ and $\Gamma_{\mathrm{PEC}}$ through the measurements and by substituting their values into the equation, the reflection coefficient of the radio wave absorber can be derived.

(6) $$\begin{array}{ll} \Gamma_{\mathrm{Total}} & =\Gamma_{1}-T_{1}T_{2}\Gamma_{3}+T_{1}T_{2}\Gamma_{2}\Gamma_{3}^{2}-T_{1}T_{2}\Gamma_{2}^{2}\Gamma_{3}^{3}+\cdots \\ & =\Gamma_{1}-T_{1}T_{2}\Gamma_{3}\sum_{n=0}^{\infty }{\left(-\Gamma_{2}\Gamma_{3}^{}\right)}^{n}\\ & =\frac{\Gamma_{1}+\Gamma_{1}\Gamma_{2}\Gamma_{3}-T_{1}T_{2}\Gamma_{3}}{1+\Gamma_{2}\Gamma_{3}} \end{array}$$

(7) $$\Gamma_{3}=-\frac{\Gamma_{\mathrm{MUT}}-\Gamma_{\mathrm{Air}}}{\Gamma_{\mathrm{PEC}}-\Gamma_{\mathrm{Air}}}\times \frac{1-\Gamma_{\mathrm{PEC}}\Gamma_{\mathrm{Air}}}{1-\Gamma_{\mathrm{MUT}}\Gamma_{\mathrm{Air}}}$$

**Figure 6:**
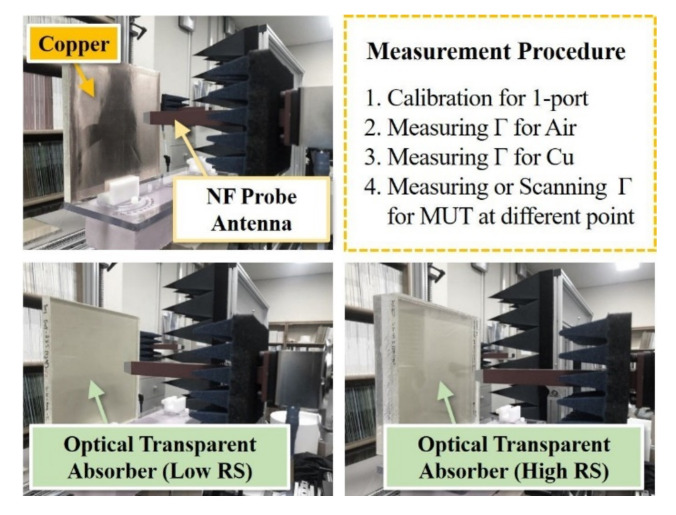
The experimental setup and procedure for the feasibility study of the single near-field antenna-based measurement.

To verify the effectiveness of the reflection coefficient measurement method using a single near-field antenna, transparent absorbing structures with $\Gamma_{\mathrm{Air}}$ and $\Gamma_{\mathrm{Copper}}$, and different thicknesses and surface resistances were fabricated (size 150 mm × 150 mm, thickness 10 mm and 30 mm). To obtain the reflection coefficient $\Gamma_{\mathrm{MUT}}$, the experimental environment shown in Figure 6 was constructed. To observe only the signal completely reflected by the surface, a copper sheet was attached to the back of the transparent absorbing structure and was measured.

**Figure 7:**
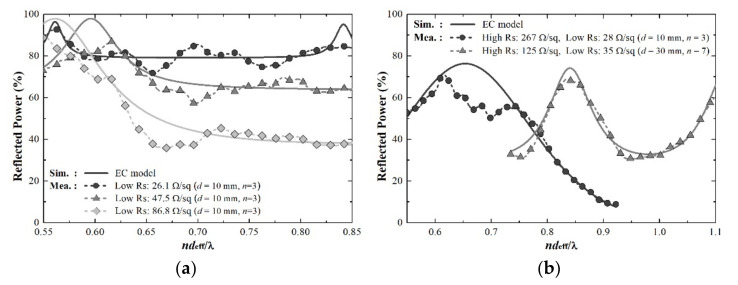
Measurement results of reflectivity for optical transparent absorbers that have different thickness and surface resistance: (**a**) single-side (low Rs) coated acrylic and (**b**) double-side (high Rs/low Rs) coated acrylic.

Figure 7 shows the reflected power result ($P_{reflec}={\left|\Gamma_{3}\right|}^{2}\times 100\%$) obtained from Equation (7). The measurement results confirmed a difference of up to 1.46 dB for the reflection coefficient and up to 4.68% for the X-band average reflectance. This is a probable measurement error caused by multiple reflected fields from the sample because of the nature of the open-ended near-field antenna, which can only facilitate measurement at a close distance. Nevertheless, the results demonstrated that the changing trends of the reflection coefficient were similar and that the errors in the reflection coefficient and average reflectance were small. Table 1 summarizes the measurement results in detail.

### 3.2. Design and Validation of Defect Diagnosis Analysis Method

**Figure 8:**
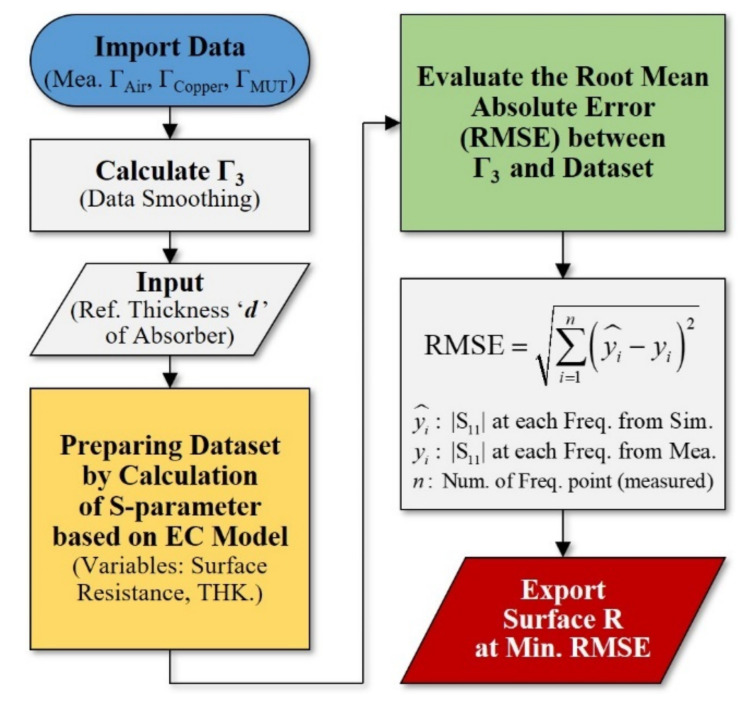
Procedure for surface resistance prediction of an optical transparent absorber based on the near-field antenna measurement method.

As mentioned above, the detection of the defects that can occur when fabricating and operating a transparent RAS, as well as the evaluation of their effects, are considered to be essential for the practical application of the proposed design. On the basis of the reflection coefficient measured by the near-field antenna and by comparing the analysis results of the equivalent circuit model using a regression evaluation metric, the surface resistance of the resistive surface (coating layer) can be predicted, and the effects on the defect area and electromagnetic performance can be analyzed from the predicted change in surface resistance. In this study, a procedure was designed for predicting the surface resistance of the resistive surface of a transparent RAS, as shown in Figure 8.

Based on the measurement results of the transparent RAS discussed in Section 3.1, the predicted surface resistance was compared with the surface resistance values measured with a non-contact sheet resistance meter (Napson Co. EC-80, Typ. accuracy of ±0.5%). Table 1 also shows the measurement error of the reflection characteristic evaluation system constructed using a single near-field antenna. A Salisbury absorbing structure with a size of 250 mm × 250 mm and a thickness of 22.5 mm was fabricated. A change in surface resistance was observed at 100 points at 11 mm intervals in a 110 mm × 110 mm high-resistance surface region in the center of the sample. To build a dataset for the root mean absolute error (RMSE) evaluation, the surface resistance and substrate thickness were assumed to be variables that play a major role in predicting the surface resistance, and the reflection coefficient was calculated according to the changes in the surface resistance of 150–700 Ω/sq and with a thickness of 22–23 mm using the equivalent circuit model.

**Figure 9:**
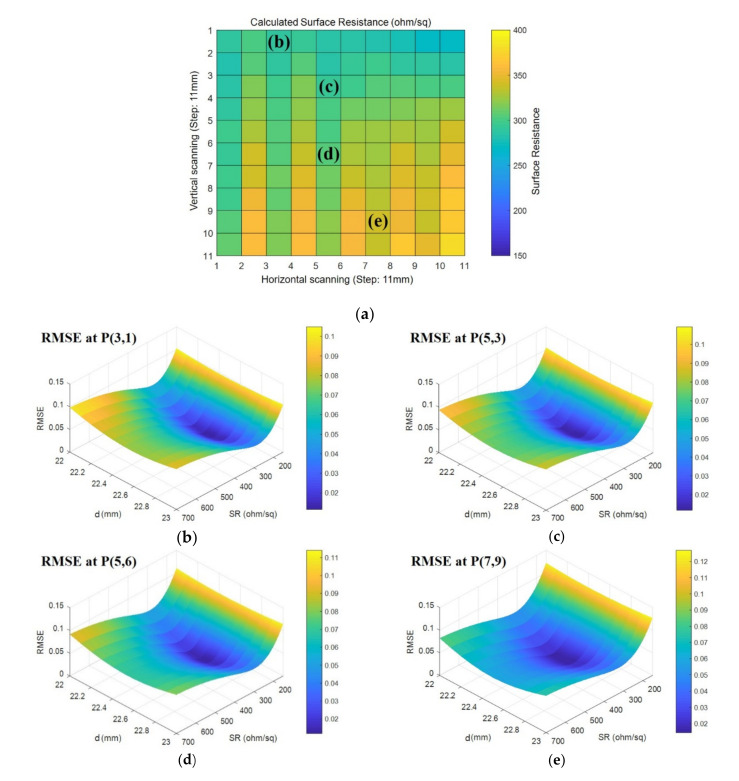
Surface resistance prediction results based on an evaluation of the root mean square error (RMSE) with calculations and measurements of reflectivity: (**a**) surface resistance distribution for a high RS on the absorber, (**b**) RMSE at P(3,1), (**c**) RMSE at P(5,3), (**d**) RMSE at P(5,6), and (**e**) RMSE at P(7,9).

Figure 9a shows the predicted surface resistance distribution for each point, and Figure 9b–e compares the surface resistance prediction process at four randomly selected points, as well as the analysis and measurement results of the reflection coefficient at each point. As shown in Figure 9b–e, the minimum value of RMSE was determined by comparing the reflection coefficient calculated according to the changes in surface resistance and thickness with the measurements for each point. The surface resistance value used in the analysis was derived as the predicted value. A comparison of the reflection coefficient values according to the change in frequency demonstrated that the values were consistent. From the above experimental results, the surface resistance of a specific region in a transparent RAS could be predicted in order to detect defects. Furthermore, the presence of defects and their influence could be analyzed through the point at which considerable changes in the surface resistance are observed.

## 4. Conclusions

To reduce the electromagnetic wave interference due to the cavity resonance or electromagnetic wave leakage, in this study, an RAS with a high visible light transmittance was designed and fabricated based on an transparent conductor. Furthermore, for practical applications, in order to detect defects that could occur during the fabrication and operation, and to analyze their effects, a system based on an open-ended near-field antenna was constructed for evaluating the reflection characteristics, a defect analysis procedure was designed, and the effectiveness of the design was verified.

The proposed defect analysis procedure can quickly evaluate the local electromagnetic performance of planar transparent and three-dimensional absorption structures. Accordingly, based on its ability to detect physical defects, the design is expected to be applicable in the nondestructive testing field. The key contributions of this study are as follows.

I. A radio wave absorber with a high visible light transmittance (85.3% measured by Hazemeter) was designed, and the reflection/transmission and absorption characteristics of a sample fabricated via DC magnetron sputtering, which allowed for large-area (250 mm × 250 mm) fabrication, using a transparent conductor (indium-oxide-based TCO), were evaluated. The results confirmed a radio wave blocking of 97% and an absorption performance of 98.1% (Figure 4).
II. To detect locally occurring defects in the transparent RAS and to analyze their influence on the electromagnetic performance, a reflection coefficient measurement system using a single near-field antenna was constructed, and a surface resistance prediction algorithm based on the equivalent circuit model was designed, whose effectiveness was also experimentally verified (Figure 8 and Figure 9).

## Figures and Tables

**Table 1:** Comparisons for reflectivity between prediction and measurement.

TCO Coated Samples			Reflection Coefficient Difference	Reflected Power Difference
Thickness (d)	Measurement	Prediction		
10 mm	26.1 Ω/sq	22.0 Ω/sq	0.51 dB	0.83%
10 mm	47.5 Ω/sq	42.0 Ω/sq	0.82 dB	2.64%
10 mm	86.8 Ω/sq	89.0 Ω/sq	1.41 dB	2.99%
10 mm	267 Ω/sq	232 Ω/sq	1.47 dB	4.68%
30 mm	125 Ω/sq	110 Ω/sq	0.81 dB	1.47%

## Data Availability

Not applicable.
